# Identification and characterisation of Gamma-herpesviruses in zoo artiodactyla

**DOI:** 10.1186/s12985-024-02311-3

**Published:** 2024-02-23

**Authors:** Laura Bianchessi, Edmund Flach, Giulia Monacchia, Mark Dagleish, Madeleine Maley, Lauretta Turin, Mara Silvia Rocchi

**Affiliations:** 1 Department of Veterinary Medicine and Animal Sciences, University of Milan, Via dell’Università 6, 26900 Milan, Italy; 2 Wildlife Health Services, Zoological Society of London (retired), Regents Park, NW1 4RY London, UK; 3 CIRM Italian Malaria Network, University of Perugia, Perugia, Italy; 4 Division of Veterinary Pathology, Public Health and Disease Investigation, School of Biodiversity, One Health and Veterinary Medicine, University of Glasgow, 464 Bearsden Road, G61 1QH Glasgow, UK; 5 Moredun Research Institute, Pentlands Science Park, Bush Loan, EH26 OPZ Penicuik, UK

**Keywords:** Herpesvirus, Malignant catarrhal fever, Artiodactyla, DNA polymeras, Pan-herpes consensus PCR, Virus surveillance, Phylogenetic analyses

## Abstract

**Background:**

Viruses within the γ-herpesviruses subfamily include the causative agents of Malignant Catarrhal Fever (MCF) in several species of the order *Artiodactyla*. MCF is a usually fatal lymphoproliferative disease affecting non-adapted host species. In adapted host species these viruses become latent and recrudesce and transmit during times of stress or immunosuppression. The undetected presence of MCF-causing viruses (MCFVs) is a risk to non-adapted hosts, especially within non-sympatric zoological collections. This study investigated the presence of MCFVs in six different zoological collections in the UK, to evaluate the presence of subclinical/latent MCFVs in carrier animals.

**Methods:**

One-hundred and thirty eight samples belonging to 54 different species of *Artiodactyla* were tested by Consensus Pan-herpes PCR. The positive samples were sequenced and subjected to phylogenetic analyses to understand their own evolutionary relationships and those with their hosts.

**Results:**

Twenty-five samples from 18 different species tested positive. All viruses but one clustered in the γ-herpesvirus family and within the Macavirus as well as the non-Macavirus groups (*caprinae* and *alcelaphinae*/*hippotraginae* clusters, respectively). A strong association between virus and host species was evident in the Macavirus group and clustering within the *caprinae* group indicated potential pathogenicity.

**Conclusion:**

This study shows the presence of pathogenic and non-pathogenic MCFVs, as well as other γ-herpesviruses, in *Artiodactyla* species of conservation importance and allowed the identification of new herpesviruses in some non-adapted species.

**Supplementary Information:**

The online version contains supplementary material available at 10.1186/s12985-024-02311-3.

## Introduction

Herpesviruses belong to a large family of DNA viruses known as *Herpesviridae*, which includes several pathogens of humans and other animals including mammals, reptiles, birds, amphibians, fish and molluscs. Globally distributed, herpesviruses can cause a range of infections from clinically inapparent to severe. Generally, herpesviruses adapt to their host species and, if a host survives the initial primary infection, they frequently establish a latent, persistent subclinical infection where the virus remains dormant in the host’s cells and may reactivate under certain conditions. Viral recrudescence due, for example, to immunosuppression, stress, concurrent infection and other factors can result in severe disease in the host species. Herpesvirus infections can have serious consequences, often presenting as abortion in pregnant dams or as a spectrum of disease in foetuses and neonates [1]. Some herpesviruses can cross species barriers through spillover events infecting non-adapted hosts in which they cause, typically, fatal clinical diseases [2]. Transmission is through direct contact with infected individuals, including oral, genital or, in some cases, airborne transmission [3].

Herpesviruses have a characteristic structure composed of a linear, double-stranded deoxyribonucleic acid (DNA) genome, protected by an icosahedral capsid, surrounded by a proteinaceous matrix (tegument) and associated with a glycoprotein-containing lipid envelope [1]. The herpesvirus genome is characterised by high stability, but it is also capable of changing rapidly in response to evolutionary selection pressures. This may lead to new viral strains or species that could adapt to new hosts [4]. Based on biological and genetic characteristics, along with current molecular phylogenetic analyses, the family *Herpesviridae* is divided into three sub-families: *Alpha (α)-herpesvirinae*, *Beta (β)-herpesvirinae* and *Gamma (γ)-herpesvirinae* [5]. Viruses belonging to the subfamily *γ-herpesvirinae* have, subsequently, been divided into seven genera, of which the *Macavirus* genus is of particular interest in *Artiodactyla* due to the ability of some of its members to cause severe clinical disease when infecting non-host species. Based on antigenic similarities, five members of this genus (Alcelaphine γ-herpesvirus-1, Alcelaphine γ-herpesvirus-2, Ovine γ-herpesvirus-2, Caprine γ-herpesvirus-2 and Hippotragine γ-herpesvirus-1), as well as five additional non*-*Macaviruses (Ibex-MCFV, MCFV-white-tailed deer/Caprine γ-herpesvirus-3, Oryx-MCFV, Muskox-MCFV and Aoudad-MCFV) are further grouped into the Malignant Catarrhal Fever Virus (MCFV) group. Viruses in this group are characterised by the presence of a 15-A antigenic epitope in the glycoprotein B fusion protein and by high similarity in conserved amino acid sequences in the DNA polymerase enzyme [6–9]. MCFVs are named according to their highly adapted reservoir host species [9, 10] and six of these are recognised as pathogenic, causing Malignant Catarrhal Fever (MCF) in non-adapted host species (as shown in Table 1). Based on adapted-host species and phylogenetic relationships, MCFVs are also divided into two subgroups: the *Caprinae* group and the *Alcelaphine/Hippotraginae* group. A complete list of the viruses attributed to those two groups and the corresponding abbreviations, when present, is presented in Table 1 [9, 10].

**Table 1:** MCFVs, their adapted and non-adapted host species and potential to induce MCF

Virus Name	Adapted host Species	Non-adapted host Species	Clinical MCF
***Ovine γ-herpesvirus-2*** ***(OvGHV-2)***	Bighorn sheep (*Ovis canadensis*) Domesticated sheep (*Ovis aries*) Dall’s sheep (*Ovis dalli*) Mouflon (*Ovis gmelini*)	African buffalo *(Syncerus caffer)* American bison (*Bison bison*) Axis deer (*Axis axis*) Bali cattle/banteng (*Bos javanicus*) Blackbuck antelope *(Antelope cervicapra)* Cattle (*Bos taurus*) Elk *(Cervus canadensis)* European bison (*Bison bonasus)* Fallow deer *(Dama dama)* Formosan sika deer (*Cervus nippon taiouanus*) Giraffe (*Giraffa camelopardalis)* Goat *(Capra hircus*) Horse (*Equus caballus*) Malayan Sambar Deer *(Cervus unicolor)* Mule deer *(Odocoileus hemionus)* Moose (*Alces alces)* Nile lechwe *(Kobus megaceros)* Pig (*Sus scrofa*) Père David’s deer (*Elaphurus davidianus*) Reeve’s muntjac *(Muntiacus reevesi)* Red deer *(Cervus elaphus)* Roe deer (*Capreolus capreolus*) Rusa deer (*Rusa timorensis*) Sika deer (*Cervus nippon*) Water buffalo *(Bubalus bubalis)* White-tailed deer (*Odocoileus virginianus*) Zebu (*Bos indicus*)	Yes
***Caprine γ-herpesvirus-2*** ***(CpGHV-2)***	Goat (*Capra hircus*)	Moose (*Alces alces)* Pronghorn (*Antilocapra americana*) Pudu (*Pudu puda*) Roe deer *(Capreolus capreolus)* Sika deer (*Cervus nippon)* Water buffalo (*Bubalus bubalis*) White-tailed deer (*Odocoileus virginianus*)	Yes
***MCFV-white-tailed deer/Caprine γ-herpesvirus-3 MCF-WTD/CpGHV-3)***	Goat (*Capra hircus*)	Red brocket deer (*Mazama americana*) Reindeer (*Rangifer tarandus*) White-tailed deer (*Odocoileus virginianus*)	Yes
***Ibex-MCFV***	Ibex (*Capra nubiana*)	Anoa (*Bubalus depressicornis)* Black duiker (*Cephalophus niger*) Bongo antelope (*Tragelaphus eurycerus*) Pronghorn (*Antilocapra americana*) Red-flanked duiker (*Cephalophus rufilatus*) Yellow-backed duiker (*Cephalophus silvicultor*)	Yes
***Aoudad-MCFV***	Barbary sheep (or Aoudad) (*Ammotragus lervia*)	NR	
***Muskox-MCFV***	Musk ox (*Ovibos moschatus*)	NR	
Alcelaphine γ-herpesvirus-1 (AlGHV*-*1) *	Blue wildebeest (C*onnochaetes* *taurinus*) Black wildebeest (*Connochaetes gnou*)	African buffalo *(Syncerus caffer)* American bison (*Bison bison*) Bali cattle/banteng *(Bos javanicus)* Barasingha deer (*Cervus duvauceli*) Cattle (*Bos taurus*) Gaur *(Bos gaurus)* Red-flanked duiker (C*ephalophus rufilatus*) Water buffalo *(Bubalus bubalis)* Zebu (*Bos indicus*)	Yes
Alcelaphine γ-herpesvirus-2 (AlGHV-2) *	Hartebeest (*Alcelaphus buselaphus*) Topi (*Damaliscus lunatus*)	American bison (*Bison bison*) Barbary red deer (*Cervus elaphus barbarous*)	Yes
Hippotragine γ-herpesvirus-1 (HiGHV-1) *	Roan antelope (*Hippotraginae* *equinus*)	NR	
Oryx-MCFV *	Gemsbok (*Oryx gazella*)	NR	

The MCFVs belonging to the *Caprinae* group are shown in italic bold, while the MCFVs belonging to the *Alcelaphine*/*Hippotraginae* group are shown with asterisk [10, 11]. NR = no reported cases of MCF; unclassified = herpesviruses not attributed to any subfamily.

MCFVs infect many ungulate species of the order *Artiodactyla* and have also been reported in horses (*Perissodactyla*) [12]. The most well-adapted reservoir hosts belong to the *Bovidae* families, such as blue and black wildebeest, roan antelope, sheep and goats. In the adapted hosts, MCFVs establish latent infections in lymphoid tissues. During viral recrudescence, the adapted hosts can shed viruses into the environment via nasal and ocular discharges and birth products. Consequently, either through direct contact with the reservoir host or via fomites, MCFVs can infect non-adapted hosts [10]. The infected non-adapted hosts can, in some cases, develop MCF, which is an aggressive lymphoproliferative and typically fatal disease. The main clinical signs are fever, loss of appetite, profuse nasal discharge, corneal opacity, generalised lymphadenopathy, neurological compromise (tremors and convulsions due to angiocentric lymphocytic encephalitis), upper respiratory and gastrointestinal tract lesions. The affected animals typically die within a few days of the clinical signs developing, but some may survive for several weeks [10, 13, 14]. However, MCFVs cannot replicate in non-adapted hosts therefore they are dead-end hosts and cannot pass the virus on to other animals. The non-adapted hosts usually belong to the *Cervidae*, *Giraffidae, Suidae* and *Bovidae* families, as shown in Table 1 (non-adapted host species). Occasionally, both adapted and non-adapted hosts can be infected by more than one MCFV [10, 11].

Currently, the most studied MCFVs are OvGHV-2 and AlGHV-1 and only their genomes have been sequenced [15]. Therefore, the epidemiology of MCF is well defined for these two viruses only. OvGHV-2 is carried sub-clinically by domestic and wild sheep but causes sheep-associated MCF (SA-MCF) in cattle and deer. SA-MCF is present worldwide. AlGHV-1 is carried sub-clinically by blue and black wildebeest and causes wildebeest-associated MCF (WA-MCF), especially in cattle in sub-Saharan Africa and in zoological species worldwide. SA-MCF and WA-MCF can be differentiated only on an etiological and, in some cases, epidemiological basis [10, 13, 14]. The World Organisation for Animal Health (OIE) recognises histopathology as the gold standard definitive diagnosis for MCF. Brain, liver, kidney and lymphoid tissues of animals affected by MCF characteristically show vasculitis due to infiltration by lymphoid cells. However, a more accurate diagnosis is obtained by combining clinical signs, histopathology, serological or molecular methods [14, 15]. There are no commercial vaccines or treatments available for MCF. The only available control method is based on preventing interactions between adapted and non-adapted hosts, avoiding the sharing of pastures, housing and water troughs, especially during stressful situations, such as transport and parturition, when the viral shedding increases [11, 15].

The disease in Europe is generally sporadic in nature, nevertheless, when presenting as an outbreak, MCF can affect up to 40% of a cattle herd [15], in Africa, MCF is one of the most severe diseases affecting cattle populations [10], with a case fatality of WA-MCF of up to 95–100% [16], in New Zealand, MCF impacts financially on deer farms, while in the USA the disease causes severe economic loss to bison producers [10]. In zoological collections, MCF represents a serious risk to the conservation of endangered species kept for breeding programs. Few MCF studies have been carried out in zoos and wildlife parks, therefore, knowledge of the epidemiology of MCF in exotic species is poor [15]. Investigating the presence of MCFV in zoological collections may identify new susceptible potential hosts as well as reservoir hosts, along with the existence of potentially novel pathogenic MCFVs [17, 18]. In addition, these studies could help understand the disease risk associated with keeping groups of usually non-sympatric animals in close proximity [15]. Consequently, control of MCF in livestock and zoological collections will be improved [15, 19, 20].

The aim of this study was to investigate the presence of MCFVs in fifty-four species within the order *Artiodactyla* housed in six different zoological collections within the UK using Consensus Pan-herpes PCR. The latter is a powerful molecular tool used to detect known and unknown mammalian herpesviruses and has been used extensively to detect herpesviruses in many non-host adapted species as well as host-adapted reservoir species [21]. None of the animals investigated showed overt signs of or was suspected to have died of MCF.

## Materials and methods

### Sample Collection and DNA extraction

**Fig. 1:**
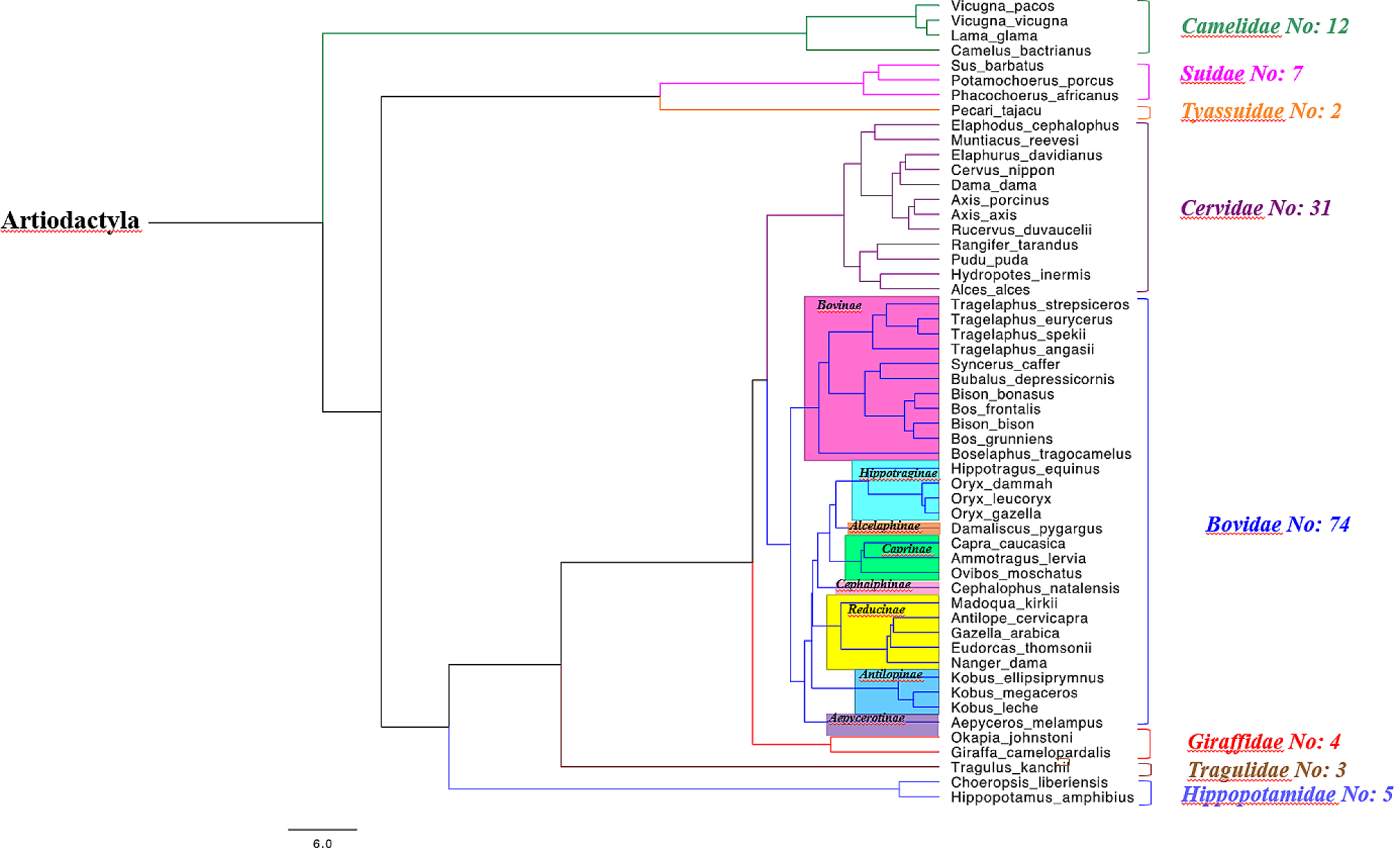
Artiodactyla species phylogenetic tree. Artiodactyla species included in the study, their phylogenetic relationships to each other and corresponding sample numbers. The phylogenetic tree was reconstructed using probabilistic inference of molecular and fossil data using the online tool VertLife ( https://vertlife.org/phylosubsets/accessed on 25th April 2023) [22] and it was visualized and edited using FigTree v1.4.4 (http://tree.bio.ed.ac.uk/software/figtree/ accessed on 25th April 2023) http://tree.bio.ed.ac.uk/software/figtree/. Distance scale represents difference between sequences in substitutions per site

One hundred and thirty-four tissue samples from fifty-four different species belonging to the order *Artiodactyla* were collected between 1997 and 2017 in six different zoological collections in the UK (Zoological Society of London; Whipsnade and London Zoos, Paighton Zoo, Twycross Zoo, Royal Zoological Society of Scotland; Edinburgh Zoo and the Highland Wildlife Park). The sampling included a maximum of three individuals per species, none of which showed the main clinical signs of MCF at the time of death nor the typical lesions during *post-mortem* examination. Figure 1 (*Artiodactyla* tree) shows the species from which the samples were collected, their phylogenetic relationship and the number of samples per family.

Two different tissue samples from each individual were selected for DNA extraction when available. Spleen and lymph node were the preferred tissues, but kidney, liver, lung, buccal mucosa or blood were used if spleen and/or lymph node were not represented. Tissue homogenates in Viral Transport Media, and buffy coats from uncoagulated blood were processed following the manufacturer’s protocols for DNA extraction using the DNeasy®blood and tissue kit (Qiagen, Hilden, Germany). DNA quality was verified using a NanoDrop one microvolume spectrophotometer (ThermoFisher Scientific, Waltham, MA, USA).

### Consensus Pan-herpesvirus PCR and sequencing of part of the DNA polymerase catalytic subunit genes

The Consensus Pan-herpesvirus PCR [21] was used to detect the presence of γ*-*herpesviruses in the samples. The PCR was carried out in a nested format with degenerate/deoxyinosine-substituted primers, as described previously [23, 24]. In brief, three primers (DFA, ILK and KG1) were used for the first-round PCR, and two primers (TGV and IYG) for the second-round PCR. The primer sequences are illustrated in Table 2.

**Table 2:** Primer sequences employed for the Consensus Pan-herpes PCR according to Ehlers et al., 1999 [24]

FIRST-ROUND PCR		
	***Forward***	***Reverse***
*Degenerate* *primers*	**DFA**: 5^’^-GAYTTYGC**N**AGYYT**N**TAYCC-3^’^ **ILK**: 5^’^-TCCTGGACAAGCAR**N**YSGC**N**MT**N**AA-3^’^	**KG1**: 5^’^-GTCTTGCTCACCAG**N**TC**N**CA**N**CCYTT-3^’^
*Deoxyinosine-* *Substituted* *equivalent* *primers*	**DFA**: 5^’^-GAYTTYGC**I**^**a**^AGYYT**I**^**a**^TAYCC-3^’^ **ILK**: 5^’^-TCCTGGACAAGCAR**I**^**a**^YSGC**I**^**a**^MT**I**^**a**^AA-3^’^	**KG1**: 5^’^-GTCTTGCTCACCAG**I**^**a**^TC**I**^**a**^CA**I**^**a**^CCYTT-3^’^
**Product 440 bp**		
**SECOND-ROUND PCR**		
	*Forward*	*Reverse*
*Degenerate* *primers*	**TGV**: 5^’^-TGTAACTCGGTGTAYGG**N**TTYAC**N**GG**N**GT-3’	**IYG**: 5^’^-CACAGAGTCCGTRTC**N**CCRTA**D**AT-3’′
*Deoxyinosine-* *Substituted* *equivalent* *primers*	**TGV**: 5^’^-TGTAACTCGGTGTAYGG**I**^**a**^TTYAC**I**^**a**^GG**I**^**a**^GT-3’	**IYG**: 5^’^-CACAGAGTCCGTRTC**I**^**a**^CCRTA**I**^**a**^AT-3’
**Product 220 bp**		

Degenerate and inosine-substituted equivalents are shown in bold. Degenerate bases are coded according to the IUPAC system [25]. I = Deoxyinosine.

First and second round-PCR reactions were carried out by using the HotStarTaq DNA polymerase® kit (Qiagen, Hilden, Germany), according to the manufacturer’s instructions, in a total volume of 25 µL containing 2 µL of purified DNA. Thermal cycling conditions were 15 min at 95 °C, followed by 45 cycles of 30 s at 94 °C, 60 s at 46 °C and 60 s at 72 °C, with a final elongation of 10 min at 72 °C. In the second round-PCR, 2 µL of the first reaction products were used in a 48 µL reaction mixture. Thermal cycling conditions were the same as the first round-PCR. PCR products were visualised on a 1.5% TAE agarose gel according to standard procedures. Amplicons were gel-purified using the ChargeSwitch™ PCR clean-up kit (Thermo Fisher) according to the manufacturer’s instructions, and Sanger sequenced bi-directionally by Eurofins MWG (https://eurofinsgenomics.eu/en/custom-dna-sequencing/eurofins-services/tubeseq-service/ accessed 15th May 2023).

### Nucleotide sequence analysis

Nucleotide sequences were analysed using BioEdit (V7.2) software [26]. Consensus sequences were generated by aligning the reverse and forward sequences, then trimmed to remove the primers. The nucleotide sequences generated in this study are shown in supplementary Fig. 1. Trimmed consensus sequences were validated using Basic Local Alignment Search Tool (BLAST) (https://blast.ncbi.nlm.nih.gov/Blast.cgi/ accessed 22nd May 2023). Validated nucleotide sequences were translated into amino acid sequences using the Expasy Translate Tool (https://web.expasy.org/translate/ accessed 23rd May 2023).

### Phylogenetic analyses

Amino acid sequences were aligned using MAFFT (https://www.ebi.ac.uk/Tools/msa/mafft/ accessed 23rd May 2023). The amino acid sequences generated in this study are shown in supplementary Fig. 1. Initial phylogenetic analysis was performed using an alignment of the seventeen amino acid sequences obtained in this study (one duplicate was removed) and forty-four reference sequences obtained from GenBank (https://www.ncbi.nlm.nih.gov/genbank/ accessed 23rd May 2023). The reference amino acid sequences used in this study are shown in supplementary Table 1. These reference sequences included one viral amino acid sequence for each genus of *α-herpesvirinae* and *β-herpesvirinae* subfamilies, and all viral amino acid sequences recognised as γ-herpesviruses by the International Committee on Taxonomy of Viruses (ICTV) (https://ictv.global/report/chapter/herpesviridae/herpesviridae/ accessed 23rd May 2023). According to the Bayesian Information Criterion (BIC), the best-fit model of amino acid substitution was LG + I + G4, which was selected by the model selection tool in IQ-TREE [27, 28]. Phylogenetic analysis was carried out on amino acid sequences as previously proposed for herpesviruses [5, 29] and due to the wide divergence of herpesvirus gene sequences across the different families [30].

The second phylogenetic analysis was carried out using an alignment of the ten amino acid sequences belonging to the genus *Macavirus* identified in the initial analysis, and thirteen DNA polymerase catalytic subunit reference amino acid sequences, including nine different viral species belonging to the *Macavirus* genus and four MCFVs [8]. The best-fit model (using BIC) of amino acid substitution was determined by the model selection tool in IQ-TREE, and it was JTT + G4.

Phylogenetic trees were generated by the maximum likelihood (ML) method in IQ-TREE [27], and the reliability of internal branches was estimated by ultrafast bootstrap (UFBoot) [27–31]. The trees were exported in Newick format to Dendroscope 3.8.4 for graphical editing [32].

## Results

### Positive samples and Virus Identification

Twenty-five animals from eighteen species were positive for herpesviruses. All PCR products produced high quality sequences with only one sequence (Herpes 19) present in two different animals of the same species (Bearded pig). The size of the trimmed sequences was between 166 and 178 bp, due to insertions and deletions reflecting viral species. The twenty-five individual positive animals are listed in Table 3 alongside the corresponding infecting viral species identified by BLAST.

**Table 3:** Host species and corresponding infecting herpesviruses

Positive Sample ID	Host Species	Closest match (BLAST Search)	Accession Number	Percentage similarity	Code (Virus ID)
Herpes 1	American bison *(Bison bison)*	Ovine γ−herpesvirus 2	EU309722.1	100%	H1
Herpes 2	Formosan sika deer *(Cervus nippon taiouanus)*	Ovine γ−herpesvirus 2	EU078708.1	100%	H2
Herpes 3	Gaur *(Bos frontalis gaurus)*	Ovine γ−herpesvirus 2	EU309722.1	100%	H1
Herpes 4	Musk ox *(Ovibos moschatus)*	Muskox rhadinovirus	MN316573.1	100%	H3
Herpes 5	Musk ox *(Ovibos moschatus)*	Muskox rhadinovirus	MN316573.1	100%	H3
Herpes 6	Common hippo *(Hippopotamus amphibius)*	Hippopotamus amphibious rhadinovirus 1	AY854170.1	100%	H4
Herpes 7	Pygmy hippo *(Choeropsis liberiensis)*	Hexaprotodon liberiensis γ−herpesvirus 1	AY197559.2	100%	H5
Herpes 8	Pygmy hippo *(Choeropsis liberiensis)*	Hexaprotodon liberiensis γ−herpesvirus 1	AY197559.2	100%	H5
Herpes 9	Impala (*Aepyceros melampus)*	Impala herpesvirus 1	AB194012.1	100%	H6
Herpes 10	Blackbuck *(Antilope cervicapra)*	Bovidae γ−herpesvirus 2	MN599419.1	100%	H7
Herpes 11	Waterbuck *(Kobus ellisiprymnus)*	Ruminantia γ−herpesvirus 3	MN599424.1	100%	H8
Herpes 12	Nile lechwe *(Kobus megaceros)*	Ruminantia γ−herpesvirus 4	MN599435.1	100%	H9
Herpes 13	Nile lechwe *(Kobus megaceros)*	Ruminantia γ−herpesvirus 4	MN599435.1	100%	H9
Herpes 14	Nile lechwe *(Kobus megaceros)*	Ruminantia γ−herpesvirus 4	MN599435.1	100%	H9
**Herpes 15**	Bearded pig (*Sus barbatus)*	Porcine lymphotropic herpesvirus 1	NC_038264.1, AF118399.1	99.44%	H10
Herpes 16	Gemsbok *(Oryx gazella)*	Hippotragine γ−herpesvirus 2	MN599437.1	99.43%	H11
Herpes 17	Scimitar-horned orix *(Oryx dammah)*	Hippotragine γ−herpesvirus 2	MN599437.1	99.43%	H11
Herpes 18	Scimitar-horned orix *(Oryx dammah)*	Hippotragine γ−herpesvirus 2	MN599437.1	99.43%	H11
**Herpes 19**	Bearded pig (*Sus barbatus)*	Porcine lymphotropic herpesvirus 1	NC_038264.1, AF118399.1	98.31%	H12
***Herpes 20***	Roan antelope (*Hippotragus equinus*)	Hippotragine γ−herpesvirus 2	MN599437.1	92.53%	H13
***Herpes 21***	Red river hog (*Potamochoerus porcus*)	Phacochoerus africanus cytomegalovirus 1	AY197558.1	82.66%	H14
***Herpes 22***	Lesser mouse deer (*Tragulus kanchil*)	Tapirus terrestris γ−herpesvirus 1	AF141887.3	76.40%	H15
***Herpes 23***	Lesser mouse deer (*Tragulus kanchil*)	Tapirus terrestris γ−herpesvirus 1	AF141887.3	75.78%	H16
*Herpes 24*	Kirk’s dik-dik *(Madoqua kirkii)*	Rusa unicolor equina γ−herpesvirus 1 Madoqua kirkii γ−herpesvirus 1	MN599444.1 MN599446.1	98.84% 98.12%	H17
Herpes 25 *	Chinese water deer (*Hydropotes inermis*)	Mustelid γ− herpesvirus 2 Mustelid γ− herpesvirus 1	MN082678.1 NC_038266.1	94.37% 89.70%	H18

BLAST analyses of sequences amplified in this study and percentage similarity to GenBank reference sequences (Accession number). Sequences with less than 97% similarity are identified in italic bold; sequences that may correspond to more than one viral species are in italic and sequences that may correspond to more than one viral strain are in bold. Sequences with less than 97% similarity to GenBank-deposited sequences and corresponding to more than one virus species are shown with asterisk. Code = name assigned in this study.

The results of the BLAST analysis identified 18 unambiguous partial herpesvirus DNA polymerase catalytic subunit nucleotide sequences with variable percentages of identity to sequences deposited in GenBank. Of these, 12 were assigned by homology to the gamma herpesvirus sub-family, whereas six matched non-gamma herpesviruses.

Nine of these sequences were identical (100%) to reference sequences (coded H1 to H9), while four revealed a percentage similarity between 98% and 99% (H10-12 and H17). The sequences in italic bold and with asterisks in Table 3 (H13-H16) showed a percentage similarity to reference sequences of less than 97%, with only one (H13) having the virus species matching the host species. Some sequences, indicated in italic and with asterisks in Table 3, belonging to the Kirk’s dik-dik and the Chinese water deer samples (H17 and H18, respectively), could be matched to more than one deposited virus sequence. The Kirk’s dik-dik sequence (H17) has a similar identity to two deposited sequences, one of which has also been detected in a Kirk’s dik-dik sample; albeit the percentage coverage is higher for the Rusa unicolor equina virus (98% and to 91%, respectively). The sequence derived from the Chinese water deer samples (H18) also had two possible matches, both of them with Mustelid herpesviruses, either 1 or 2, one with 94.37% similarity, but only 85% query coverage, and the other with 89.7% similarity, but a 99% query coverage. Finally, the sequences indicated in bold in Table 3 (H10 and H12) also matched more than one reference viral nucleotide sequence (and corresponding aminoacid sequence), with both matches belonging to the same virus species.

All the hosts belonging to the same Artiodactyla species were infected by viruses that had identical nucleotide sequences except the two bearded pig samples (H10 and H12); these generated two sequences diverging at two positions, with one representing a non-synonymous substitution. Moreover, the viral nucleotide sequences detected in the two lesser mouse deer (H15 and H16) diverged in one position, but in both cases represented a synonymous substitution. Based on the above results, only seventeen partial amino acid sequences of the herpesvirus DNA polymerase catalytic subunit were reliably identified. The sequences generated in this work have been deposited in the NCBI database under accession numbers from PP346672 to PP346689.

.

### Phylogenetic analyses

**Fig. 2:**
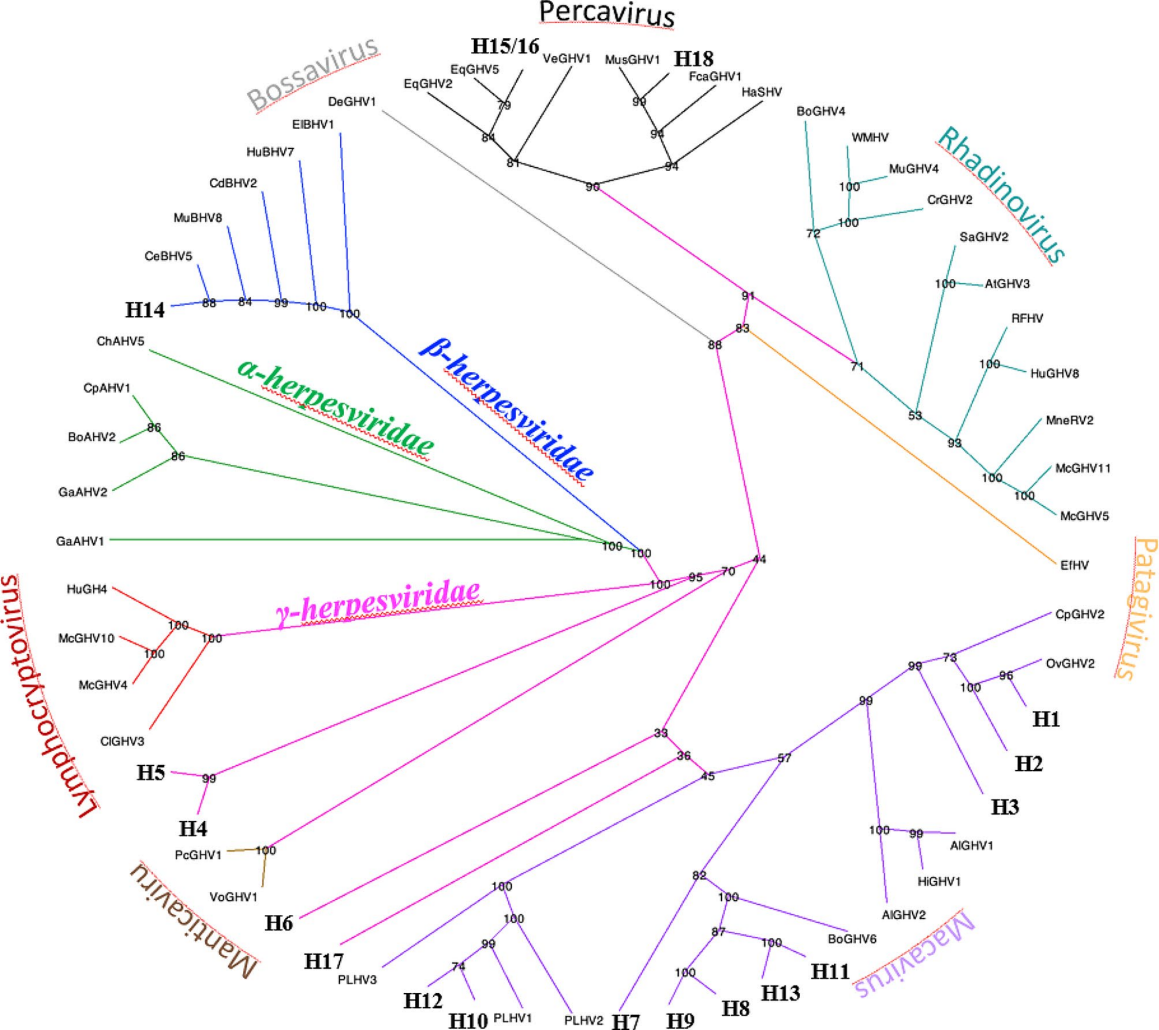
Estimation of the evolutionary relationships between the amino acid sequences of the herpesvirus DNA polymerase. The maximum likelihood phylogenetic tree presented as a circular cladogram was created by using seventeen herpesvirus partial DNA polymerase catalytic subunit translated amino acid sequences generated in this study (showed with the corresponding code number) and 44 herpesvirus DNA polymerase catalytic subunit amino acid reference sequences downloaded from GenBank. Accession numbers of the reference sequences are listed in the supplementary file Table 1. The bootstrap probability (1000 replicates) is shown next to each node. Subfamilies are represented with different colours: green (α-herpesvirinae), blue (*β-*herpesvirinae) and fuchsia (γ-herpesvirinae). Genera within the γ-herpesvirinae subfamily are also indicated by different colours: red (Lymphocryptovirus), brown (Manticavirus), purple (Macavirus), orange (Patagivirus), teal (Rhadinovirus), black (Percavirus) and grey (Bossavirus). List of abbreviations, clockwise starting from the first virus in the α-herpesvirinae subfamily: GaAHV1 (Gallid γ-herpesvirus 1), GaAHV2 (Gallid γ-herpesvirus 2), BoAHV2 (Bovine γ-herpesvirus 2), CpAHV1 (Caprine γ-herpesvirus 1), ChAHV5 (Chelonid γ-herpesvirus 5), CeBHV5 (Cercopithecine γ-herpesvirus 5), MuBHV8 (Murid γ-herpesvirus 8), CdBHV2 (Caviid γ-herpesvirus 2), HuBHV7 (Human γ-herpesvirus 7), ElBHV1 (Elephantid γ-herpesvirus 1), DeGHV1 (Common bottlenose dolphin γ-herpesvirus 1), EqGHV2 (Equid γ-herpesvirus 2), EqGHV5 (Equid γ-herpesvirus 5), VeGHV1 (Vespertilionid γ-herpesvirus 1), MusGHV1 (Mustelid γ-herpesvirus 1), FcaGHV1 (Felis catus γ-herpesvirus 1), HaSHV (Harp seal herpesvirus), BoGHV4 (Bovine γ-herpesvirus 4), WMHV (Wood mouse herpesvirus), MuGHV4 (Murid γ-herpesvirus 4), CrGHV2 (Cricetid γ-herpesvirus 2), SaGHV2 (Saimiriine γ-herpesvirus 2), AtGHV3 (Ateline γ-herpesvirus 3), RFHV (Retroperitoneal fibromatosis-associated herpesvirus), HuGHV8 (Human γ-herpesvirus 8), MneRV2 (Macaca nemestrina rhadinovirus 2), McGHV11 (Macacine γ-herpesvirus 11), McGHV5 (Macacine γ-herpesvirus 5), EfHV (Eptesicus fuscus γ-herpesvirus), CpGHV2 (Caprine γ-herpesvirus 2), OvGHV2 (Ovine γ-herpesvirus 2), AlGHV1 (Alcelaphine γ-herpesvirus 1), HiGHV1 (Hippotragine γ-herpesvirus 1), AlGHV2 (Alcelaphine γ-herpesvirus 2), BoGHV6 (Bovine γ-herpesvirus 6), PLHV2 (Porcine lymphotropic herpesvirus 2), PLHV1 (Porcine lymphotropic herpesvirus 1), PLHV3 (Porcine lymphotropic herpesvirus 3), VoGHV1 (Vombatid γ-herpesvirus 1), PcGHV1 (Phascolarctid γ-herpesvirus 1), ClGHV3 (Callitrichine γ-herpesvirus 3), McGHV4 (Macacine γ-herpesvirus 4), McGHV10 (Macacine γ-herpesvirus 10) and HuGH4 (Human γ-herpesvirus 4)

An initial phylogenetic analysis was undertaken to clarify the evolutionary relationships between the seventeen herpesvirus amino acid sequences derived from the 18 nucleotide sequences identified in this study and the forty-four reference amino acid sequences currently recognised by the International Committee on Taxonomy of Viruses (ICTV) as herpesviruses. The phylogenetic tree is shown in Fig. 2.

As estimated by the phylogenetic analysis, the herpesviruses identified in this study, except for H14 which corresponds to Phacochoerus africanus cytomegalovirus 1 and belongs to the *β-herpesvirinae* sub-family, have phylogenetic relationships with viruses recognised as γ-herpesviruses, despite the lack of attribution to this sub-family when using GenBank reference sequences (see above). In this sub-family, H15/16 and H18 cluster with viruses situated in the *Percavirus* genus, although for all of them the low identity or the ambiguity suggests that the identity of these viruses still needs to be confirmed. H1, H2 and H3 cluster with Ovine γ -herpesvirus 2 (OvGHV-2, *Macavirus* genus). Sequences H7, H8, H9, H11 and H13 cluster with Bovine γ-herpesvirus 6 (BoGHV-6), whereas H10 and H12 cluster with three Porcine lymphotropic γ-herpesviruses (PLHV-1, PLHV-2 and PLHV-3) in the *Macavirus* genus. With the sequences available, the phylogenetic analysis was not able to assign a close relationship for H4, H5, H6 and 17 and other herpesvirus sequences.

**Fig. 3:**
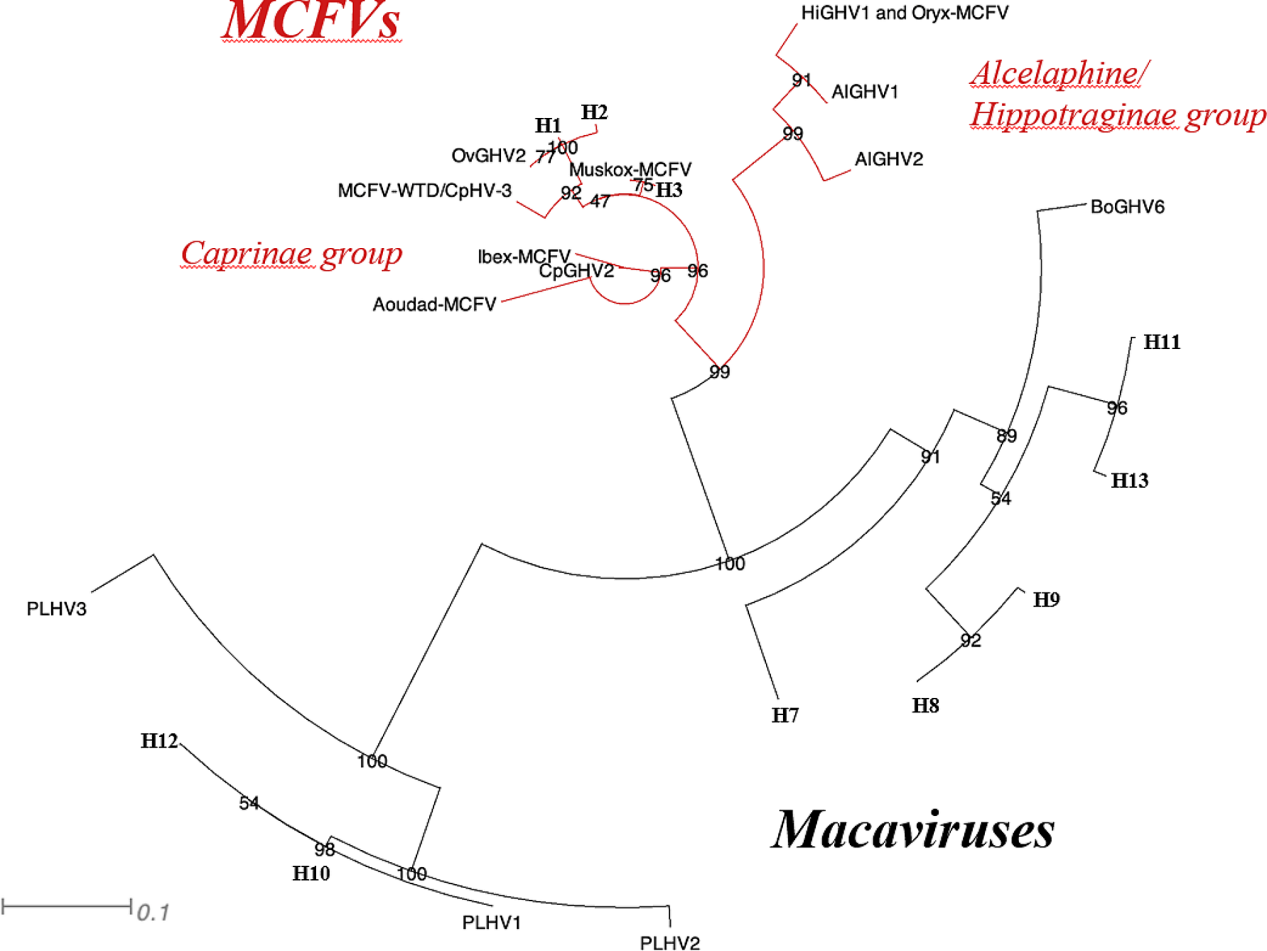
Evolutionary relationships of amino acid sequences of the herpesviruses DNA polymerase of Macaviruses and MCFVs. The MCFV group includes both Macaviruses and non-Macaviruses. The maximum likelihood phylogenetic tree, presented as a circular phylogram, was generated from an alignment of the ten herpesvirus partial DNA polymerase catalytic subunit amino acid sequences produced in this study (H1, H2, H3, H7, H8, H9, H10, H11, H12 and H13) with thirteen Macavirus and MCFVs DNA polymerase catalytic subunit amino acid reference sequences obtained from GenBank. The bootstrap probability for clades was obtained from 1000 replicates and is shown next to each node. Distance scale represents difference between sequences in substitutions per site. Macavirus and MCFVs are represented with different colours: black (Macavirus) and dark purple (MCFVs). HiGHV-1 (Hippotragine γ-herpesvirus 1) and MCFV-oryx have identical partial DNA polymerase amino acid sequences [9]. Abbreviations: Aoudad-MCFV (Aoudad-Malignant Catarrhal Fever Virus), CpGHV2 (Caprine γ-herpesvirus 2), Ibex-MCFV (Ibex-Malignant Catarrhal Fever Virus), MCFV-WTD/CpHV3 (Malignant Catarrhal Fever Virus-White-tailed deer/Caprine herpesvirus 3), OvGHV2 (Ovine γ-herpesvirus 2), Muskox-MCFV (Muskox-Malignant Catarrhal Fever Virus), HiGHV1 (Hippotragine γ-herpesvirus 1), Oryx-MCFV (Oryx-Malignant Catarrhal Fever Virus), AlGHV1 (Alcelaphine γ-herpesvirus 1), AlGHV2 (Alcelaphine γ-herpesvirus 2), BoGHV6 (Bovine γ-herpesvirus 6), PLHV2 (Porcine lymphotropic herpesvirus 2), PLHV1 (Porcine lymphotropic herpesvirus 1) and PLHV3 (Porcine lymphotropic herpesvirus 3)

An additional phylogenetic analysis was performed to investigate the evolutionary relationships among ten of the herpesvirus amino acid sequences identified as Macaviruses (H1, H2, H3, H7, H8, H9, H10, H11, H12 and H13) and reference sequences including those of the genus *Macaviruses* as well as non-*Macavirus* MCFVs. The phylogenetic tree is shown in Fig. 3.

The phylogenetic tree shows the clustering of the MCFVs into two different sub-lineages belonging to the *Caprinae* group and *Alcelaphine/Hippotraginae* groups. Three of the herpesviruses in the *Caprinae* group, specifically H1 and H2, appear closely related to OvGHV-2, while H3 shows similarity to Muskox-MCFV. The other seven herpesviruses identified in this study cluster within the *Macavirus* genus. Within this genus, the clustering seems to reflect the host-virus relationship, since H10 and H12, found in bearded pigs, cluster with Porcine lymphotropic herpesvirus 1, 2 and 3, whereas H7, 8, 9, 11 and 13 are divided into subclusters which mimics their host evolutionary relationship (e.g.: H7 and H8 both belong to *Antilopinae* hosts whereas H11 and H13 to *Hippotraginae*).

## Discussion

Climate change, population displacement and the need for more land for food production are some of the factors that currently endanger wildlife [33]. Conservation of endangered wildlife species held within zoological collections aims to retain genetic diversity through selective breeding programmes and leads to the exchange of animals between collections [34]. However, the distribution of γ-herpesviruses in captive wildlife is currently not well understood and individual animals, if not screened pre-movement, can become the source of outbreaks of MCF.

Initial screening identified twenty-five samples from animals belonging to eighteen different *Artiodactyla* species that tested positive for herpesviruses. Due to the ambiguity of the sequences, in terms of viral genus and species assignment, when compared to reference sequences deposited in GenBank, phylogenetic analysis to confirm or negate this initial identification was undertaken. Using sequence and phylogenetic analyses, all but one of the viruses present were attributed to the *γ-herpesvirinae* sub-family, with the exception of the virus identified as Phacochoerus africanus cytomegalovirus 1, which belongs to the *β-herpesvirinae* sub-family.

The nucleotide sequences detected in American bison, Formosan sika deer and gaur were all identical to OvGHV-2 reference sequences, with the American bison and gaur matching the same reference sequence, while the Formosan sika deer matched a different reference sequence; both sequences also clustered phylogenetically with OvGHV-2 in the *Caprinae* MCF group, confirming their identity. SA-MCF disease has been documented previously in these three animal species, especially American bison and Formosan sika deer, which are known to be highly susceptible hosts to OvGHV-2 [11, 35–39]. Therefore, the presence of this virus is consistent with contact with an ovine species, either domesticated sheep, mouflon, Bighorn or Dall’s sheep. In contrast, our identification of OvGHV-2 in a gaur represents only the second report in the literature [40] as this species is usually affected by AlGHV-1 [41, 42], and confirms the ability of OvGHV-2 to infect this host species.

The sequences recovered from the two musk oxen were identical to a Muskox rhadinovirus sequence deposited in GenBank. This relationship was confirmed by phylogenetic analysis where this virus was shown to cluster within the *Caprinae* MCFV group. As reported previously, musk oxen are considered host-adapted to the Muskox rhadinovirus, although there are no reports of this virus causing MCF in susceptible species [9, 43].

The sequences obtained from the common hippo and the pygmy hippo returned sequences identical to the γ-herpesviruses found previously in their respective host species: Hippopotamus amphibious rhadinovirus 1 and Hexaprotodon liberiensis gammaherpesvirus 1 [44]. Phylogenetic analysis showed that these γ-herpesviruses are evolutionarily very close, having derived from the same ancestor. However, they do not belong to any known *γ-herpesvirinae* genera and they are not part of the MCFVs. These *Artiodactyla* species were recognised as adapted hosts of their own herpesviruses by previous studies [44], but there is no information about these viruses’ pathogenicity in other animals. Similarly, the impala carried its own species-specific herpesvirus, Impala herpesvirus 1, which had been identified previously in clinically healthy free-ranging impala in South Africa [45]. Phylogenetic analysis showed that this virus does not belong to any known γ-herpesvirus genera, and it is not closely related to MCFVs. Currently, it is not known whether this virus is pathogenic in other animal species.

The blackbuck carried a γ-herpesvirus and the sequence was identical to a Bovidae γ-herpesvirus 2 reference sequence; this virus was previously identified in this species and in a Cretan wild goat (*Capra hircus cretica*, also called the kri-kri) in San Diego Zoo [19]. However, there are no other documented cases of MCF or another disease caused by this virus, and the only previously reported case of MCF in blackbucks was attributed to Ovine γ-herpesvirus 2 [46]. This apparent discrepancy could be explained by the San Diego Zoo study assigning provisional names to new DNA polymerase catalytic subunit sequences according to the host family in which the virus was found if it did not have a 100% similarity to previously reported GenBank reference sequences [19]. Therefore, the name *bovidae* indicates a generic group of viruses with multi-species tropisms. Our phylogenetic analysis indicates that Bovidae γ-herpesvirus 2, despite diverging from other viruses in the same genus, is still located in the *Macavirus* cluster, therefore it should be classified as non-MCFV virus of unknown pathogenicity.

Herpesvirus sequences identified in the waterbuck and the Nile lechwe showed 100% identity with Ruminantia γ-herpesvirus 3 and 4, respectively. These virus names were generated by the same San Diego Zoo study described above [19]. Ruminantia γ-herpesvirus 3 and Ruminantia γ-herpesvirus 4 were also found in other clinically healthy host species of the suborder *Ruminantia* such as domestic cattle, ellipse waterbuck, scimitar-horned oryx, Indian sambar (*Rusa unicolor unicolor*), Javan rusa (*Rusa timorensis russa*), Addax (*Addax nasomaculatus*), Nile lechwe, Ugandan kob (*Kobus kob thomasi*) and Indian axis deer [19]. This suggests that waterbuck and Nile lechwe might be adapted hosts of these viruses as all the samples tested in this study were from animals not suspected of MCF clinically or upon post-mortem examination. A case of SA-MCF due to Ovine γ-herpesvirus 2 has also been reported in this species [47], thus the Nile lechwe is probably a carrier of its own herpesvirus, as are most species, but also susceptible to MCF by infection with other species of herpesviruses [19]. Our phylogenetic analyses indicated that both the waterbuck and Nile lechwe sequences belong to the *Macavirus* genus, in the non-MCFV cluster, and they show a high probability of a common ancestor. However, the sequence from the waterbuck identified as Ruminantia γ-herpesvirus 3 had a 99% similar identity (one nucleotide difference, representing a synonymous substitution) with a sequence deposited previously in GenBank corresponding to the Macavirus strain wasserbock virus. This virus was found previously in a waterbuck and it is considered to be a γ-herpesvirus adapted to this *Artyodactyla* species [18]. Due to the low sequence coverage, it is not possible to say if the viral sequence detected in the waterbuck represents the Macavirus strain wasserbock virus or the Ruminantia γ-herpesvirus 3 as a longer sequence and additional data generated from other Ruminantia species are required to achieve this discrimination.

A similar uncertainty occurred in assigning virus identity to the sequences identified in the gemsbok and scimitar-horned oryx, despite both showing a very high similarity to Hippotragine gammaherpesvirus 2. The study by Partin et al., 2021 in San Diego Zoo detected Hippotragine gammaherpesvirus 2 sequence in healthy animals belonging to the *Hippotraginae* sub-family, specifically in the addax and Arabian oryx (*Oryx leucoryx*), with their sequences showing 99.40% similarity to the reference strain Type 2 ruminant rhadinovirus of addax [19]. The same sequence also showed a very high similarity to Type 2 ruminant rhadinovirus of addax and Type 2 ruminant rhadinovirus in oryx, viruses previously found in addax, gemsbok, and scimitar-horned oryx species [48]. Thus, gemsbok and scimitar-horned oryx might be host-adapted to the Type 2 ruminant rhadinovirus in oryx or the Hippotragine gammaherpesvirus 2. Our phylogenetic analyses showed that the sequences identified in the gemsbok and the scimitar-horned oryx have a closer genetic relationship with the viruses belonging to the *Macavirus* genus in the *non-MCFV* cluster. There is little information regarding the pathogenicity of Ruminantia γ-herpesviruses 3 and 4, Hippotragine γ-herpesvirus 2 or Type 2 ruminant rhadinovirus in oryx in susceptible hosts other than a γ-herpesvirus isolated from scimitar-horned oryx and inoculated into a rabbit in which caused typical clinical signs and histological lesions of MCF. However, this γ-herpesvirus was not sequenced [18] therefore the pathogenicity of these viruses in the corresponding host species remains unknown.

Porcine lymphotropic herpesvirus 1 was detected in bearded pigs. This is the only case where different nucleotide and amino sequences were found in two animals belonging to the same species, both with very high similarities to two different reference sequences. Phylogenetic analyses indicated that their viral amino acid sequences belong to the *Macavirus* genus and are closely related to Porcine lymphotropic herpesvirus 1 and Porcine lymphotropic herpesvirus 2, and more distantly related to Porcine lymphotropic herpesvirus 3. Currently, there are no reports of these viruses causing clinical disease, however, as they are evolutionarily distant from viruses belonging to the MCFV group, such as Ovine γ-herpesvirus 2 and Alcelaphine γ-herpesvirus 1, they might represent non-pathogenic host-adapted viruses of domestic and wild pigs that could become pathogenic in a non-adapted hosts [49]. Importantly, as the xenozoonotic potential of the viruses cannot be excluded, allogenic bone marrow transplantations studies should include screening for these viruses due to Porcine lymphotropic herpesvirus 1 causing a post-transplantation lymphotropic disorder (PTLD) in minipigs [50].

In a few species, such as roan antelope, red river hog and lesser mouse deer, we identified sequences with less than 97% similarity to GenBank-deposited reference sequences. A γ-herpesvirus sequence clustering within the *Macavirus* genus was detected in the roan antelope sample; this virus has a phylogenetic relationship with virus sequences found in other animal species belonging to the *Hippotraginae* sub-family, such as gemsbok and scimitar-horned oryx [48]. Roan antelopes are known to carry Hippotragine gammaherpesvirus 1 sub-clinically [9], as well as a more recently described pathogenic γ-herpesvirus named Alcelaphine γ-herpesvirus 3 [51]. The virus sequence we detected in this species clusters with sequences such as Ruminantia γ-herpesviruses 3 and 4, which still have uncertain classifications, as discussed above. Therefore, the virus detected in the roan antelope in this study may represent a new γ-herpesvirus carried by this host species.

The red river hog sample contained a viral sequence with limited similarity to GenBank reference sequences belonging to the *β-herpesvirinae* sub-family; the same result was obtained in our phylogenetic analysis where this sequence can be clearly seen clustering with viruses of the *γ-herpseviridae* sub-family. This result could represent a discrepancy in sequences due to PCR or sequencing errors or be a new undescribed herpesvirus for this host species. Similar to other instances described above, without longer sequence data and additional reference sequences is not possible to give a clear interpretation.

The two viral sequences detected in the lesser mouse deer were closely related to Equine γ-herpesvirus 5 in the *Percavirus* genus. Equine γ-herpesvirus 5 can lead to equine multinodular pulmonary fibrosis (EMIPF) in horses, its adapted host [52]. Percaviruses are members of the γ -herpesvirus sub-family, but they have a distant evolutionary relationship with Macaviruses and other MCF viruses. Other members of the *Percavirus* genus include, as well as equine, bat, felid, mustelid and phocid herpesviruses. Viruses in this cluster do not display any common host specificity [53]. Therefore, the sequences found in lesser mouse deer could represent new herpesviruses for this host species or cluster with the other Percaviruses due to their low sequence homology. The likelihood of detecting two different sequences in the same species suggests this may be a new, undescribed virus.

The viral nucleotide sequence detected in the Kirk’s dik-dik samples showed high similarity to two different virus sequences, Rusa unicolor equina γ-herpesvirus 1 and Madoqua kirkii γ-herpesvirus 1. These sequences were described in the study by Partin et al., 2021 in San Diego Zoo and, as they failed to show high similarity to any viral sequence present in GenBank, were assigned provisional names [19]. Rusa unicolor equina γ-herpesvirus 1 was found in Malayan sambar (*Rusa unicolor equina*), while the Madoqua kirkii γ-herpesvirus 1 was found in the Cavendish’s dik-dik (*Madoqua kirkii cavendishi*) which belongs to *Madoqua kirkii* species [19], supporting the hypothesis that the sequence detected in this study might be Madoqua kirkii γ-herpesvirus 1. Currently, no cases of disease associated with this virus have been reported, nor are there any reported cases of MCF in Kirk’s dik-dik.

Our phylogenetic analyses showed that the virus sequence recovered from the Kirk’s dik-dik is not closely related to MCFVs and it does not belong to known *γ*-herpesvirus genera.

In the Chinese water deer, a viral nucleotide sequence with high (94.7%) similarity to Mustelid γ-herpesvirus 2 and a lower (89.70%) similarity to Mustelid γ-herpesvirus 1 was detected, however, the percentage coverage was higher for Mustelid γ-herpesvirus 1. The viral nucleotide sequence detected in this study clusters within the *Percavirus* genus and may represent a newly identified virus. Mustelid γ-herpesvirus 1 commonly infects the European badger (*Meles meles*) and is generally sub-clinical. However, it can recrudesce in the genitals causing sexually transmittable infections (STIs) which are associated with infertility in this species [54].

From this study, we can infer that the American bison, Formosan sika deer and gaur were affected by MCF not detected at post-mortem examination. Blackbuck and Kirk’s dik-dik may be adapted-host species of the respective viruses identified, and the clinical disease that led to the deaths of the animals from which the samples were derived could have been caused by non-infectious disease or other pathogens. Multiple herpesvirus infections within an individual host have been documented [19] and it has been demonstrated that the Consensus Pan-herpes PCR used in this study may not always detect herpesvirus in herpesvirus co-infected samples [21, 55]. Chinese water deer may be an adapted-host of a new virus belonging to the *Percavirus* genus, and its death might be explained by an unrelated disease or undetected pathogens as hypothesised for the blackbuck and Kirk’s dik-dik. Interestingly, species that we would have expected to be carrying infection tested negative, like the Kafue Flats lechwe (*Kobus leche*), Barbary sheep and East Caucasian tur (*Capra cylindricornis* or *Capra caucasica*). This could be due to an insufficient number of individuals tested, especially for the Barbary sheep.

This study confirms the utility of the Consensus Pan-herpes PCR targeting the DNA polymerase catalytic subunit gene which can be used to determine the presence of known and unknown herpesviruses and their evolutionary relationships. Although the Consensus Pan-herpes PCR is used widely by many research groups and is one of the few tools available for investigating herpesviruses in wildlife, there is an urgent necessity for additional and longer sequence analyses and the creation of a more comprehensive database of reference strains to enable virus identification with more certainty. Notwithstanding the power of whole genome approaches, the absence of reference genomes for the wildlife species described in the study makes this approach extremely difficult. Phylogenetic analyses in association with sequence analyses are also required to better identify and classify herpesviruses present in wildlife species.

## Conclusion

This study demonstrated the presence of pathogenic and non-pathogenic MCFVs, and other known γ-herpesviruses within *Artiodactyla* species in zoological collections and identified some herpesviruses in these host species for the first time. Currently, it is not known whether most γ-herpesviruses detected in this study are pathogenic. However, as they are related to pathogenic viruses the possibility remains and further studies are required to determine this.

We and others have demonstrated the utility of the DNA polymerase gene for the initial identification and characterisation of herpesviruses, however, the DNA polymerase sequences amplified by the Consensus Pan-herpes PCR are small in size (166–178 bp). Future studies require longer herpesvirus DNA sequences which will improve virus classification, phylogenetic analysis and help to determine if herpesvirus co-infection is a feature in wildlife species.

### Electronic supplementary material

Below is the link to the electronic supplementary material.

Supplementary Material 1

Supplementary Material 2

Supplementary Material 3

Supplementary Material 4

## Data Availability

No datasets were generated or analysed during the current study.
