# Supplementary material for: Identification and characterisation of Gamma-herpesviruses in zoo artiodactyla
Source: Virol J. 2024 Feb 23;21:49. doi: 10.1186/s12985-024-02311-3 (PMC10893651; doi:10.1186/s12985-024-02311-3)
Supplement: Supplementary file 1 — Supplementary Material 1 [file 12985_2024_2311_MOESM1_ESM.pdf]

CLUSTAL O(1.2.4) multiple sequence alignment

```
H14     CGTGCATGGTCTGTTGCCGTGTCTCCCCCTGGCGGCCTCGATCACCAGCATAGGGAGGGA   60
H18     GTCTTCAGGTATTTTACCATGTTTAAAAATAGCAGAAACTATAACATATGAAGGGCGACG   60
H15     ATCCACGGGGATTCTTCCGTGCATAAGAATAGCAGAGACCATCACCTATCAGGGCAGGGC   60
H16     ATCCACGGGGATTCTTCCGTGCATAAGAATAGCAGAGACCATCACCTATCAGGGCAGGGC   60
H10     TGCATCTGGTATGCTGCCCTGTCTCAAGATTGCAGAGACCATAACTATGCAAGGAAGGGC   60
H12     TGCATCTGGTATGCTGCCCTGTCTCAAGATTGCAGAGACCATAACTATGCAAGGAAGGGC   60
H6      GGCAAGTGGGATTTTACCTTGCTTAAAAATAGCCGAGACAGTTACTTTACAGGGGAGAAC   60
H17     GGCCACAGGGCTGCTACCGTGCCTGAAGATCGCGGAAACGGTGACCCTACAGGGAAGGAC   60
H3      GGCATCGGGCATGCTGCCGTGCCTCATGATAGCAGAAACAGTCACCCTGCAGGGCAGAAC   60
H1      AGCCTCCGGCATGCTGCCCTGCCTCATGATAGCCGAGACCGTGACTCTCCAGGGCCGAAC   60
H2      AGCCTCCGGCATGCTGCCCTGCCTCATGATAGCCGAGACCGTGACTCTCCAGGGCCGAAC   60
H7      GGCGTCCGGGCTGCTCCCGTGCCTTAAAATCGCCGAAACTGTCACCCTGCAGGGGCGTAC   60
H11     AGCTTCCGGCTTACTTCCGTGCCTGAAGATTGCTGAAACGGTAACGTTACAGGGGCGAAC   60
H13     AGCTTCAGGTCTACTTCCATGCCTGAAGATTGCTGAAACGGTAACATTACAGGGTAGAGT   60
H5      GGCCTCGGGCCTGCTGCCCTGCCTCAAGATCGCCGAGACGGTGACCCTGCAGGGGCGGAG   60
H4      GGCCTCGGGCCTGCTGCCCTGCCTCAAGATTGCCGAGACGGTGACCCTGCAGGGGCGGAG   60
H9      GGCCTCGGGCCTGCTGCCCTGCCTCAAGATAGCCGAGACGGTGACGCTGCAGGGGCGAAC   60
H8      GGCGTCGGGTCTGCTGCCCTGCCTCAAGATAGCTGAGACGGTGACGCTGCAGGGGCGAAC   60
               **  *  * ** **  *     * ** *   *  * **       **  *

H14     CATGCTCAGACAGACGAGTGATTTTATAAACAATGTTTTGTCGTCTAAGCGGTACATGAT   120
H18     CATGTTAGAAAAGTCTAAAAATTTTATAGAAAATATTACTCCTGTTGATATTGAAAGAAT   120
H15     CATGCTTGAAATGACTAAAAAATACATTGAAGCTCTAACATTGGAAAATTTAAGGTTCAT   120
H16     CATGCTTGAAATGACTAAAAAATACATTGAAGCTCTAACATTGGAAAATTTAAGGTTCAT   120
H10     CATGTTGGAAAAGACAAAAGTATTTGTAGAGAATTTAAGTCATGAGGATCTCCATTCTAT   120
H12     CATGTTGGAAAAGACAAAAGTATTTGTAGAGAATTTAAGTCATGAGGATCTCCATTCTAT   120
H6      AATGTTAGACAAGACTAAGCAGTTTATAGAGGGCGTGTCGCTGCCACACCTGCGTGAAAT   120
H17     CATGCTCGAGCGCACGCGCCGGTTCGTGGAGGCCATGGACACCGACGACCTGCGCCGCTT   120
H3      CATGCTGGAGAGGACCAAGCAGTTTGTGGAAAATGTGGACATCCAGTACCTACAACAGAT   120
H1      CATGTTGGAGAAGACAAAACAGTATGTGGAAAATCTGGACGTCCAGAGCCTACAGCAGAT   120
H2      CATGTTGGAGAAGACAAAACAGTTTGTGGAAAATCTGGACGTCCAGAGCCTACAGCAGAT   120
H7      AATGCTGGAAAAAACAAAACAGTACGTGGAGGGATTAAGTCGCGCAGATGTAGAAAAAAT   120
H11     CATGCTTGAAAAGACCAAACACTATGTGGAGAAACTGCAACTGACAGACTTGGAAAAAAT   120
H13     CATGCTTGAAAAGACCAAACACTATGTTGAGAGACTGCAACTGACAGACCTGGAAAAAAT   120
H5      CATGCTGGAGAGGACCAAAAACTTTGTGGAGCCCCTCACGGGCGCGGACATTGAGCGCCT   120
H4      CATGCTAGAGAGGACCAAAAACTTTGTGGAGCCCCTCACGGGCGAGGACCTTGAGCGCCT   120
H9      CATGCTGGAGAGGACCAAGCACTACGTGGAGGGCCTGCGCCAAGCTGACCTAGAGACCAT   120
H8      CATGCTAGAGAGGACCAAGCACTACGTGGAGGGGCTGCGCCAAGCTGACCTGGAGACCAT   120
         *** *        *       *   *  *     *                       *

H14     GGAGAGATTTGATTTGTCGGATAGTGATTTTCA---GGGGGATTTTTCACTGAATGTG   175
H18     TATACACAGGCCAGTAAATTGTGATTATGGTGCTAACT------------TTAGGGTT   166
H15     GCTACAGAAGCCCCTGGCCCACGTGAAGGAGGC---ATCC--------TTTCAGGTT-   166
H16     GCTGCAGAAGCCCCTGGCCCACGTGAAGGAGGC---ATCC--------TTTCAGGTT-   166
H10     CTGTAAGGTTGGCTTTATGCCTCAGTCACCAAACAGCATTGATAAACCCTTCAAGGTG   178
H12     CTGTAAGGTTGGCTTTATGCCTCAGTCACCAAACARCATTGATAARCCCTTCAAGGTG   178
H6      ATGTGGTGACCCTATATCCGAGCTAAATGGCAC---ACCCGATGCGCATTTCAGAGTG   175
H17     GTGCGGGGAGCCGCTGGCGGAGCTGGGCGCGGA---CCCGCCTCCGCGGTTCAAGGTA   175
H3      ATGCCCAAGCTCCACTATAACAAGCCTGCCGCAGCACCCAAACCCAAGGTTCACGGTT   178
H1      ATGTCCAACCCAGACTCTAAAAATTCACGCGCAGCACCCGACCCCGAGATTCACAGTG   178
H2      ATGTCCAACCCAGACTCTGAAAGTTCACGCGCAGCACCCGACCCCGAGATTCACAGTG   178
H7      TTGCAACTTTCCAGTTCCTGCCGTGCTGGGATG---CGACGACCCTCAGTTCCGCGTG   175
H11     TTGCCAACGACCGGTGCGTGCTATAGATGGTCA---CCCTAATCCAAGTCTCAATGTA   175
H13     TTGCCAGCGACCGGTGCGTGCTATTGATGGTCA---CCCTAATCCAAGTCTCAATGTA   175
H5      GTGCCAGCGGCCGGTGCCCCACGAGCCCGACGC---CTCGCTCAAGGTC---------   166
H4      GTGCCAGCGACCCGTGCCTCGCGAGCCCGACGC---CTCGCTCAAGGTC---------   166
H9      CTGCCAGAAGCCCGTGCCGGTCGCCGACGACCA---CCCTAACCCGGGTCTCCACGTG   175
H8      CTGCCAAAAGCCCGTGCCGGCCGCGGACGACCA---CCCAAACCCAGGTCTCCACGTG   175
```

Supplementary Figure 1. Multiple nucleotide sequence alignment of the partial catalytic subunit of the Herpesviruses DNA polymerase generated in this study. The alignment was created using Clustal Omega-Multiple Sequence Alignment (https://www.ebi.ac.uk/Tools/msa/clustalo/ accessed on 22nd May 2023).
