# Supplementary material for: Identification and characterisation of Gamma-herpesviruses in zoo artiodactyla
Source: Virol J. 2024 Feb 23;21:49. doi: 10.1186/s12985-024-02311-3 (PMC10893651; doi:10.1186/s12985-024-02311-3)
Supplement: Supplementary file 3 — Supplementary Material 3 [file 12985_2024_2311_MOESM3_ESM.pdf]

Supplementary Figure and Table legends

Figure S1. Multiple nucleotide sequence alignment of the partial catalytic subunit of Herpesvirus DNA polymerase generated in this study. The alignment was created using Clustal Omega-Multiple Sequence Alignment (https://www.ebi.ac.uk/Tools/msa/clustalo/accessed on 22nd May 2023).

Supplementary Figure 2. Multiple amino acid sequence alignment of the partial catalytic subunit of the Herpesviruses DNA polymerase generated in this study. The alignment was created using Clustal Omega-Multiple Sequence Alignment (https://www.ebi.ac.uk/Tools/msa/clustalo/accessed on 22nd May 2023).

Supplementary Table 1. Reference amino acid sequences used for the amino acid alignment in this study. These reference sequences included one partial or complete viral amino acid sequence for each genus of *α-herpesvirinae* and *β-herpesvirinae* subfamilies, and all viral amino acid sequences recognised as *γ*-herpesviruses by the International Committee on Taxonomy of Viruses (ICTV) (https://ictv.global/report/chapter/herpesviridae/herpesviridae/accessed 23rd May 2023).
