# Supplementary material for: Identification and characterisation of Gamma-herpesviruses in zoo artiodactyla
Source: Virol J. 2024 Feb 23;21:49. doi: 10.1186/s12985-024-02311-3 (PMC10893651; doi:10.1186/s12985-024-02311-3)
Supplement: Supplementary file 4 — Supplementary Material 4 [file 12985_2024_2311_MOESM4_ESM.pdf]

```
CLUSTAL O(1.2.4) multiple sequence alignment


H14             VHGLLPCLPLAASITSIGRDMLRQTSDFINNVLSSKRYMMERFDLS-DSDFQGDFSLNV     58
H15/16          STGILPCIRIAETITYQGRAMLEMTKKYIEALTLENLRFMLQKPLAHVK----EASFQV     55
H3              ASGMLPCLMIAETVTLQGRTMLERTKQFVENVDIQYLQQICPSSTITSLPQHPNPRFTV     59
H1              ASGMLPCLMIAETVTLQGRTMLEKTKQYVENLDVQSLQQICPTQTLKIHAQHPTPRFTV     59
H2              ASGMLPCLMIAETVTLQGRTMLEKTKQFVENLDVQSLQQICPTQTLKVHAQHPTPRFTV     59
H10             ASGMLPCLKIAETITMQGRAMLEKTKVFVENLSHEDLHSICKVGFMPQSPNSIDKPFKV     59
H12             ASGMLPCLKIAETITMQGRAMLEKTKVFVENLSHEDLHSICKVGFMPQSPNXIDXPFKV     59
H18             SSGILPCLKIAETITYEGRRMLEKSKNFIENITPVDIERIIHRPVN-CDYG---ANFRV     55
H17             ATGLLPCLKIAETVTLQGRTMLERTRRFVEAMDTDDLRRLCGEPLA-ELGADPPPRFKV     58
H6              ASGILPCLKIAETVTLQGRTMLDKTKQFIEGVSLPHLREICGDPIS-ELNGTPDAHFRV     58
H4              ASGLLPCLKIAETVTLQGRSMLERTKNFVEPLTGEDLERLCQRPVP-REP---DASLKV     55
H5              ASGLLPCLKIAETVTLQGRSMLERTKNFVEPLTGADIERLCQRPVP-HEP---DASLKV     55
H7              ASGLLPCLKIAETVTLQGRTMLEKTKQYVEGLSRADVEKICNFPVP-AVLGCDDPQFRV     58
H8              ASGLLPCLKIAETVTLQGRTMLERTKHYVEGLRQADLETICQKPVP-AADDHPNPGLHV     58
H9              ASGLLPCLKIAETVTLQGRTMLERTKHYVEGLRQADLETICQKPVP-VADDHPNPGLHV     58
H11             ASGLLPCLKIAETVTLQGRTMLEKTKHYVEKLQLTDLEKICQRPVR-AIDGHPNPSLNV     58
H13             ASGLLPCLKIAETVTLQGRVMLEKTKHYVERLQLTDLEKICQRPVR-AIDGHPNPSLNV     58
                  *:***: :* ::*  ** **  :  ::: :        :                : *
```

Supplementary Figure 2. Multiple amino acid sequence alignment of the partial catalytic subunit of the Herpesviruses DNA polymerase generated in this study. The alignment was created using Clustal Omega-Multiple Sequence Alignment (https://www.ebi.ac.uk/Tools/msa/clustalo/ accessed on 22nd May 2023).
